# Supplementary material for: Development of Woolly Hair and Hairlessness in a CRISPR−Engineered Mutant Mouse Model with KRT71 Mutations
Source: Cells. 2023 Jul 5;12(13):1781. doi: 10.3390/cells12131781 (PMC10341341; doi:10.3390/cells12131781)
Supplement: Supplementary file 1 [file cells-12-01781-s001.zip › Supplementary Figures.pdf]

Supplement Figures

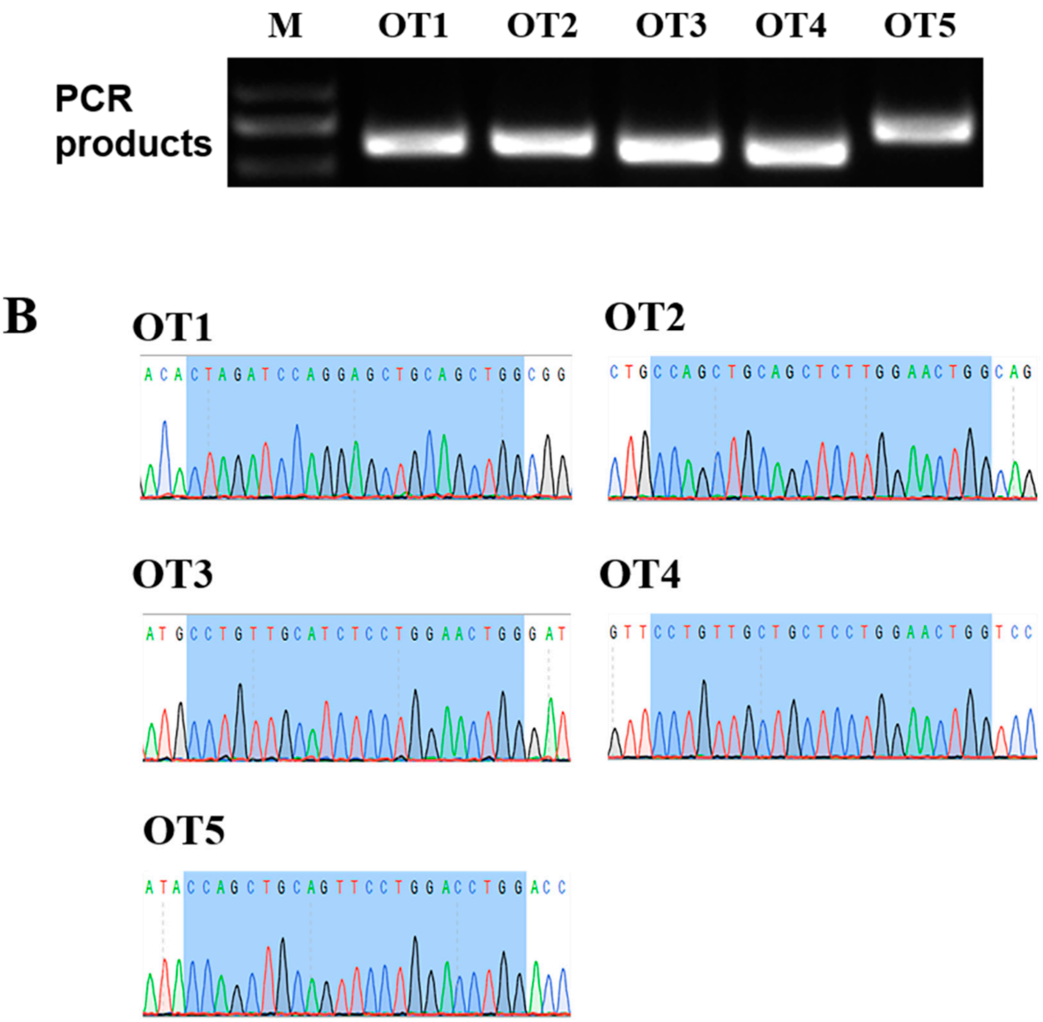

Figure S1 Off-target analysis of Krt71 in Krt71-KO mice by sanger sequencing.

(A) POTs detection by PCR in pub 2; M, D2000.

(B) Chromatogram sequence analysis of POTs using PCR products for the *Kt71* site. A total of 20 bp of the POTs and the PAMs are highlighted.

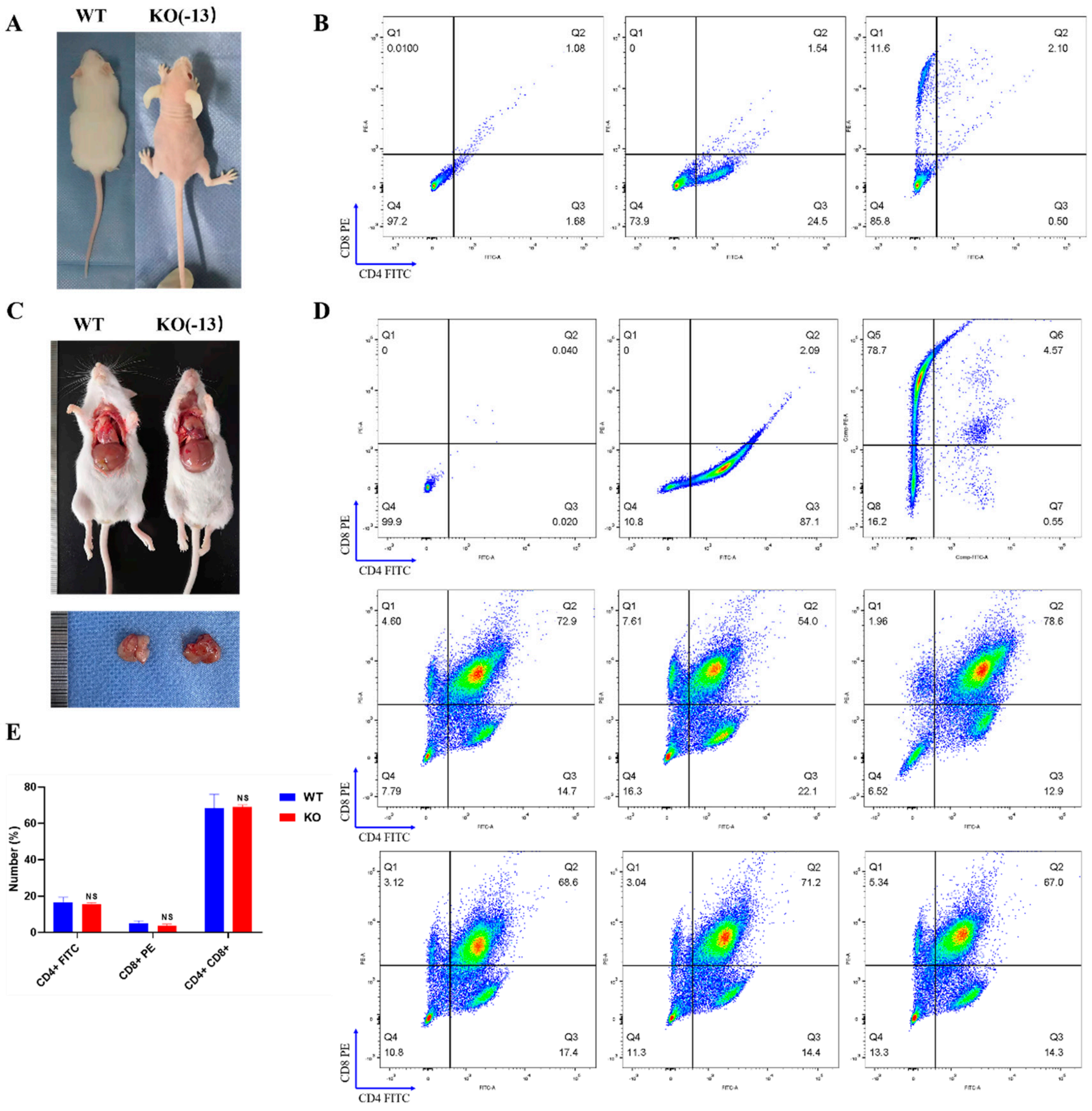

Figure S2 Thymic development analysis in Krt71-KO mice

(A) Schematic diagram of 4-weeks-old F2 Krt71-KO mice and WT mice by photo.

(B) Flow cytometry results of peripheral blood lymphocytes in WT and Krt71-KO mice.

(C) The thymus maps of WT and Krt71-KO mice in 12-weeks-old.

(D) Flow cytometry results of thymus lymphocytes in WT and Krt71-KO mice.

(E) Summary of T cell populations in thymus from three Krt71-KO mice in comparison with that from three WT mice. NS, no significance.
